# Elbow Dislocation with Complete Triceps Avulsion

**DOI:** 10.1155/2014/636504

**Published:** 2014-04-30

**Authors:** S. V. Karuppiah, D. Knox

**Affiliations:** ^1^Department of Trauma & Orthopaedics, Nottingham University Hospitals NHS Trust, Queen Medical Centre, West Block, Nottingham NG7 2UH, UK; ^2^Department of Trauma & Orthopaedics, Glasgow Royal Infirmary NHS Trust, Castle Street, Glasgow G4 0SF, UK

## Abstract

Radio-ulnar Fracture dislocation of the elbow is a high-energy trauma which can be associated with significant ligamentous injury in adults. We report an unusual triad of injury in a patient with avulsion injury of the triceps. This injury can be thought of as a variant of “terrible triad” with dislocation of radio-ulnar joint, radial head fracture, and medial collateral ligament injury with avulsion of the triceps. Elbow has to be stabilized with early repair of the ligaments for a successful outcome.

## 1. Introduction

Elbow dislocation is the second most common major joint dislocation [1]. Along with disruption of the joint capsule and restraining ligaments, accessory bony injuries are not uncommon [2]. These include fractures of the radial head and neck, capitellum, and avulsion of the coronoid process of the ulna [3].

We report an uncommon combination of injuries, posterior fracture dislocation of the elbow with complete avulsion of the distal triceps, as a variant of the terrible triad injury.

## 2. Case

A 29-year-old male patient fell off his skateboard onto an outstretched “twisting” hand sustaining an isolated closed fracture dislocation of his right elbow. On examination, there was gross swelling of the elbow and no neurovascular deficit. Radiographs confirmed posterior lateral dislocation of the elbow with fracture of the neck of the radius.

**Figure 1:**
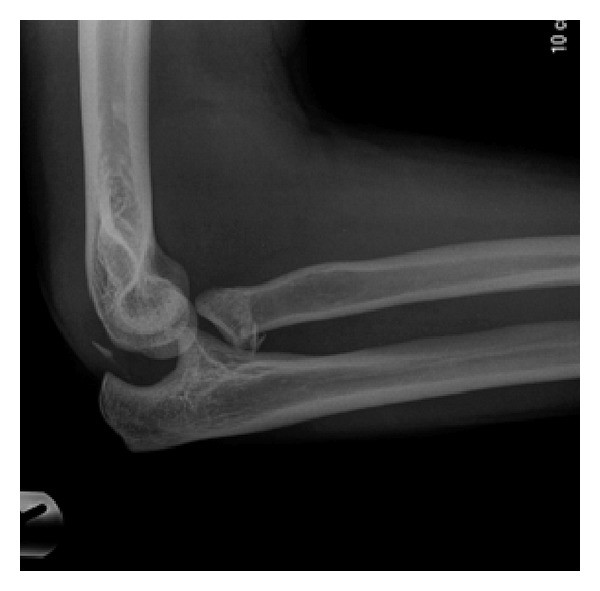
Fracture dislocation of the elbow with avulsed fragment of olecranon within the joint.

Initial reduction was attempted under sedation which proved difficult due to a small fragment of bone in the ulna humeral joint (Figure 1) and the radioulnar joint remained dislocated with the radial head sitting on top of the ulna.

The patient was taken to theatre, and the elbow was approached through a posterior incision. At this stage, extensive soft tissue damage was evident. The medial collateral ligament was torn from its distal attachment, and the triceps tendon was completely avulsed from the olecranon with a fragment of bone. The radial head was initially irreducible as it was lodged in the supinator muscle in front of the ulna.

**Figure 2:**
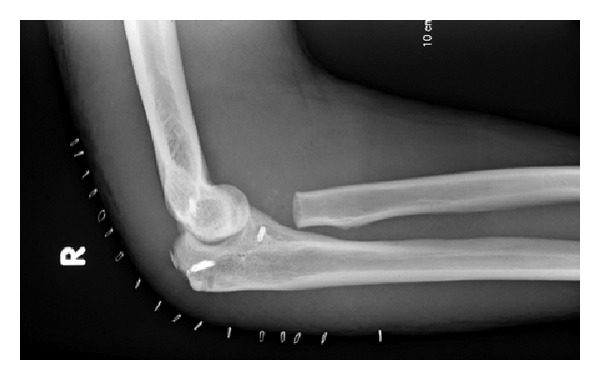
Postoperative X-ray: lateral view showing medial collateral ligament and triceps tendon reconstruction with radial head excision.

The radial head had an intra-articular fracture, and the radial head was excised. The medial collateral ligament and triceps tendon reattached with bone anchors (Mitek Surgical Products, Westwood, MA) (Figure 2). Intraoperatively, the elbow was stable after the reconstruction and did not require any radial head replacement. The elbow was immobilized in plaster and passive mobilization commenced after 4 weeks.

At 3-year followup, the patient continues to be employed in a full time job with good elbow function (Table 1). He has a good range of elbow motion enabling him to continue normal daily activities and engage in sports such as badminton and rowing.

## 3. Discussion

Posterior dislocation of the elbow is the most common presentation with anterior dislocation only accounting for less than 20% of cases [1]. Combinations of other injuries including avulsion fractures of the medial and lateral epicondyles have been reported [2, 3]. Soft tissue injuries such as rupture of the collateral ligaments can cause associated median or ulna nerve injury [3].

Several different combinations of soft tissue injuries have been described, but, to our knowledge, complete triceps rupture has not been reported before.

There are two main mechanisms that have been suggested to account for posterior dislocation of the elbow. The first theory is that the injury occurs with the force taken on the hand with the elbow fully extended. The olecranon impinges on its fossa with the force levering the ulna and radius from their capsular constraints. Then, either the brachialis muscle is torn or the coronoid process is avulsed. Continued force in extension results in ligament injury. In some instances, an abduction force will cause radial head or capitellum fracture [4].

The second theory suggests that the dislocation occurs with the elbow slightly flexed and subject to axial compressive loading. The radial collateral ligaments and lateral capsule tear allowing posterior dislocation. Depending on how the forces diverge, the radius can dislocate from the ulna [5].

Triceps tendon ruptures are rare and have previously been described in isolation. The rupture can be complete or incomplete [6]. Ruptures have been reported through different anatomical parts of the tendon, but the most common type is avulsion at the olecranon, with the most common mechanism being forced contraction of the triceps with the elbow in extension or forced extension of the elbow joint itself [7]. A fall on an outstretched hand fits this mechanism.

In our patient, the mechanism seems to be one of forced hyperextension of the elbow, resulting in the dislocation of the ulna humeral joint. A continuing abduction deforming force caused the radial head fracture, MCL tear, and a forced contraction of the triceps, whilst the elbow being in the extended position is likely to have caused the triceps rupture. There must have been a rotational force (likely forced pronation of the forearm) to cause the radial head to become embedded in the bulk of the supinator muscle.

Although the injuries seen in our patient fit with previously described mechanisms, the triceps tendon rupture associated with an elbow dislocation has not been reported before. It is clear that elbow dislocation in adults is high-energy injuries that can be associated with a variety of soft tissue injuries [8]. As described in this case, there may be part or all of the triceps tendon involved in severe injuries. If triceps rupture is not suspected, it could be missed in elbow fracture dislocations managed with a closed reduction or conservatively.

## Figures and Tables

**Table 1a:** (a) Functional evaluations (0 = best; 100 = worst)

(a) Dash disability/symptom score = 0.833 (0–100)
Work module = 0; sports/performing arts = 0
(b) Hobbies: rowing and badminton
(c) Oxford score
Pain domain = 100 (max 100)
Elbow function = 100 (max 100)
Social-psychological = 93.75 (overall 97.91) (max 300)
(d) Mayo score = 90 (excellent)

**Table 1b:** (b) Clinical evaluations

Range of movement	Right (injured side)	Left (normal side)
Flexion	130	130
Extension	40	0
Supination	30	40
Pronation	45	45
